# Severe illness as a risk factor for live burial

**DOI:** 10.1007/s12024-025-01070-z

**Published:** 2025-08-13

**Authors:** Barbara Dóra Halasi, Beáta Ágnes Borsay, Tamás Gergő Harsányi, Róbert Kristóf Pórszász, Péter Attila Gergely

**Affiliations:** Department of Forensic Medicine, University of Debrecen, Faculty of Medicine, Debrecen, Hungary

**Keywords:** Buried alive, Soil aspiration, Homicide, Obstruction of airways

## Abstract

Asphyxiation due to airway obstruction by sand is a rare occurrence, predominantly associated with accidental environmental or occupational hazards. Cases involving intentional live burial as a means of homicide are scarcely reported in forensic literature. This report presents an unusual case in which a severely ill victim was buried alive without resistance, ultimately succumbing to mechanical asphyxiation. A 32-year-old woman, suffering from perforated duodenal ulcer and peritonitis, was buried in a shallow grave inside a shed by the partner, under the mistaken belief that death had already occurred. Autopsy findings revealed pulmonary overinflation and airway obstruction by soil, confirmed through polarized light microscopy. The absence of coercive measures, alcohol, or drugs in toxicology results suggests that the victim’s critical medical condition rendered her incapable of resisting. The forensic investigation provided key evidence distinguishing live burial from post-mortem concealment, proving that death resulted from asphyxiation rather than natural disease progression. This case highlights the vital role of forensic pathology in differentiating between accidental and intentional deaths, emphasizing the need for detailed forensic analyses in cases involving potential live burial. Moreover, it raises awareness that critically ill individuals may reach a state of physical exhaustion where they cannot respond to life-threatening situations, inadvertently facilitating misjudgements that can result in fatal outcomes.

## Introduction

Asphyxia can result from external compression of the airways or from obstruction caused by a foreign substance, such as a liquid or solid. While asphyxiation is commonly associated with strangulation, suffocation, or drowning, the obstruction of airways by sand is a rare occurrence [1]. Most reported cases involve accidental entrapment in sand, often due to workplace hazards or environmental incidents, such as construction site collapses or unattended play of children where respiratory asphyxia developed due to overwhelming thoracic compression after sand burial [2] or covered beach sand hole caves [3]. Although the literature on live burial remains scarce, historical records suggest that the phenomenon was far from uncommon even in the 19th century [4]. From a forensic perspective, it is of critical importance to determine whether a victim was still alive at the time of burial, particularly in cases where the body was interred without a coffin or protective covering, such as in a plastic bag. A valuable approach involves examining the airways for the presence and extent of soil or sand consistent with the burial environment, which may indicate aspiration while still alive [5]. Similarly, in cases of live burial within a coffin, signs of attempted escape may also be detectable, for example through the identification of wood splinters beneath the victim’s fingernails and scratches on the lid of the coffin [4]. However, instances where a perpetrator intentionally buries their victim alive are rarely described in forensic literature.

The following case describes a rare instance in which the victim’s airway was obstructed by soil following live burial. The forensic findings played a crucial role in determining the circumstances of death, distinguishing between different causes of death and intentional causes, and ultimately establishing the suspect’s level of responsibility. This case highlights the importance of forensic pathology in criminal investigations, particularly in identifying key indicators of mechanical asphyxiation due to foreign material obstruction and/or chest compression by soil or sand.

## Case report

**Fig. 1:**
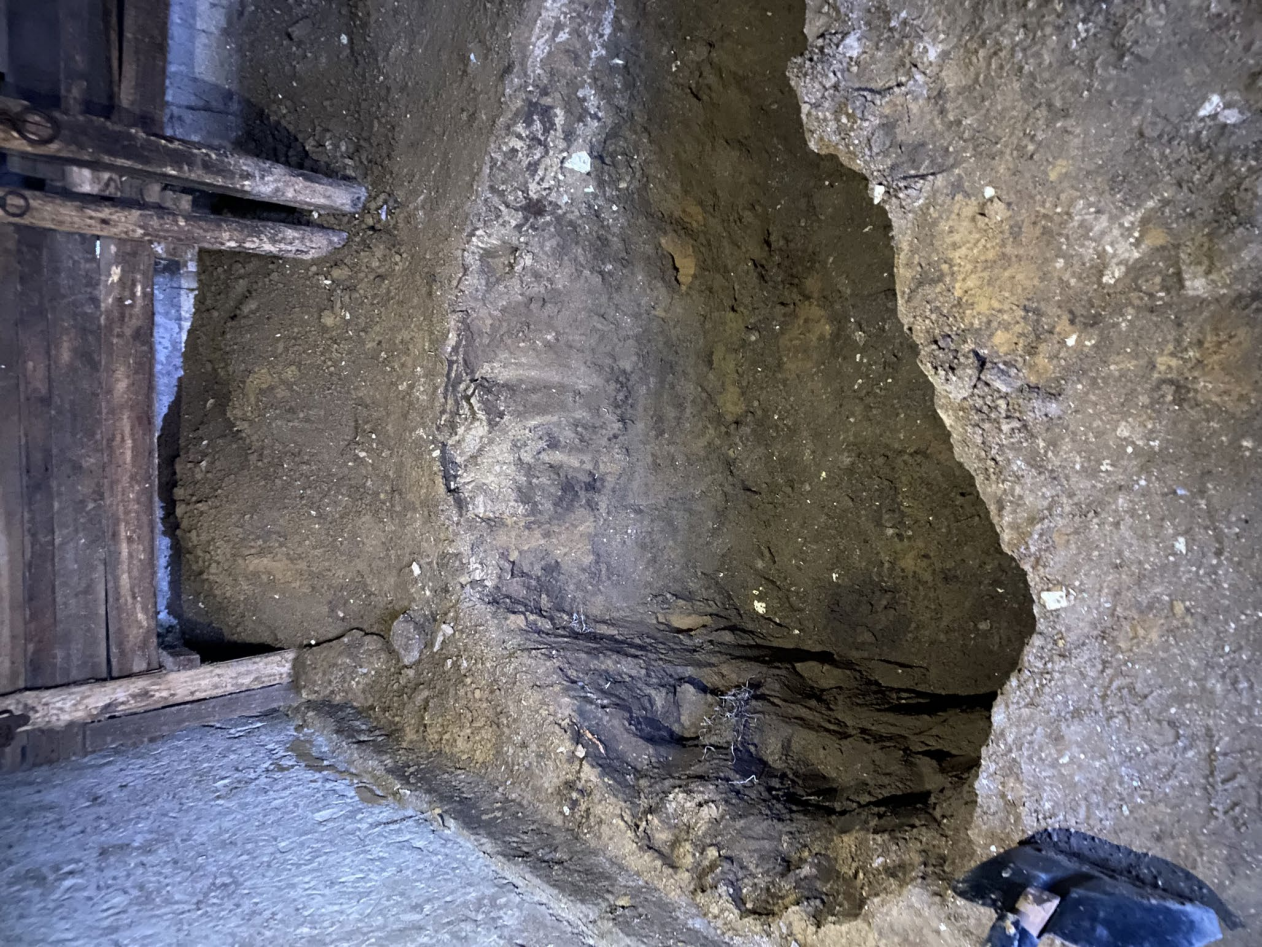
Crime Scene – Shallow Grave Inside the Shed. The shed in which the victim was buried by the perpetrator. The image shows a quite shallow hole dug into the ground. This was the location where the victim was found. The soil appears to be a mixture of clay and dirt, with loose chunks around the edges. A shovel is partially visible on the left side of the image

**Fig. 2:**
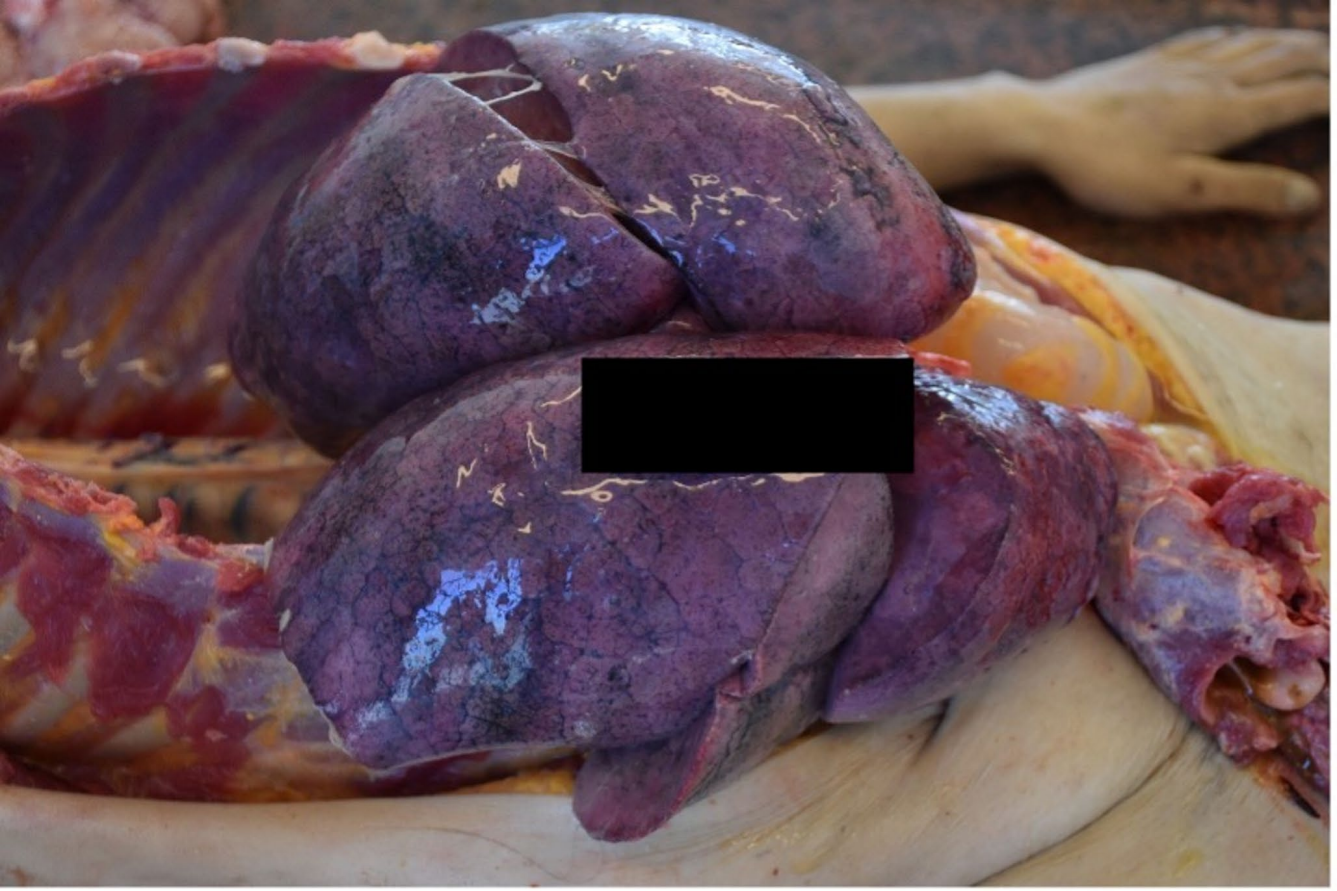
Pulmonary Overinflation Indicative of Asphyxia. Upon opening the chest cavity, we observed swollen, distended lungs. The lung tissue appeared excessively inflated, suggesting an abnormal accumulation of trapped air. This finding is consistent with pulmonary overinflation, which can result from asphyxia. The observed condition strongly suggests that the victim may have experienced oxygen deprivation, leading to respiratory distress and ultimately contributing to the cause of death

**Fig. 3:**
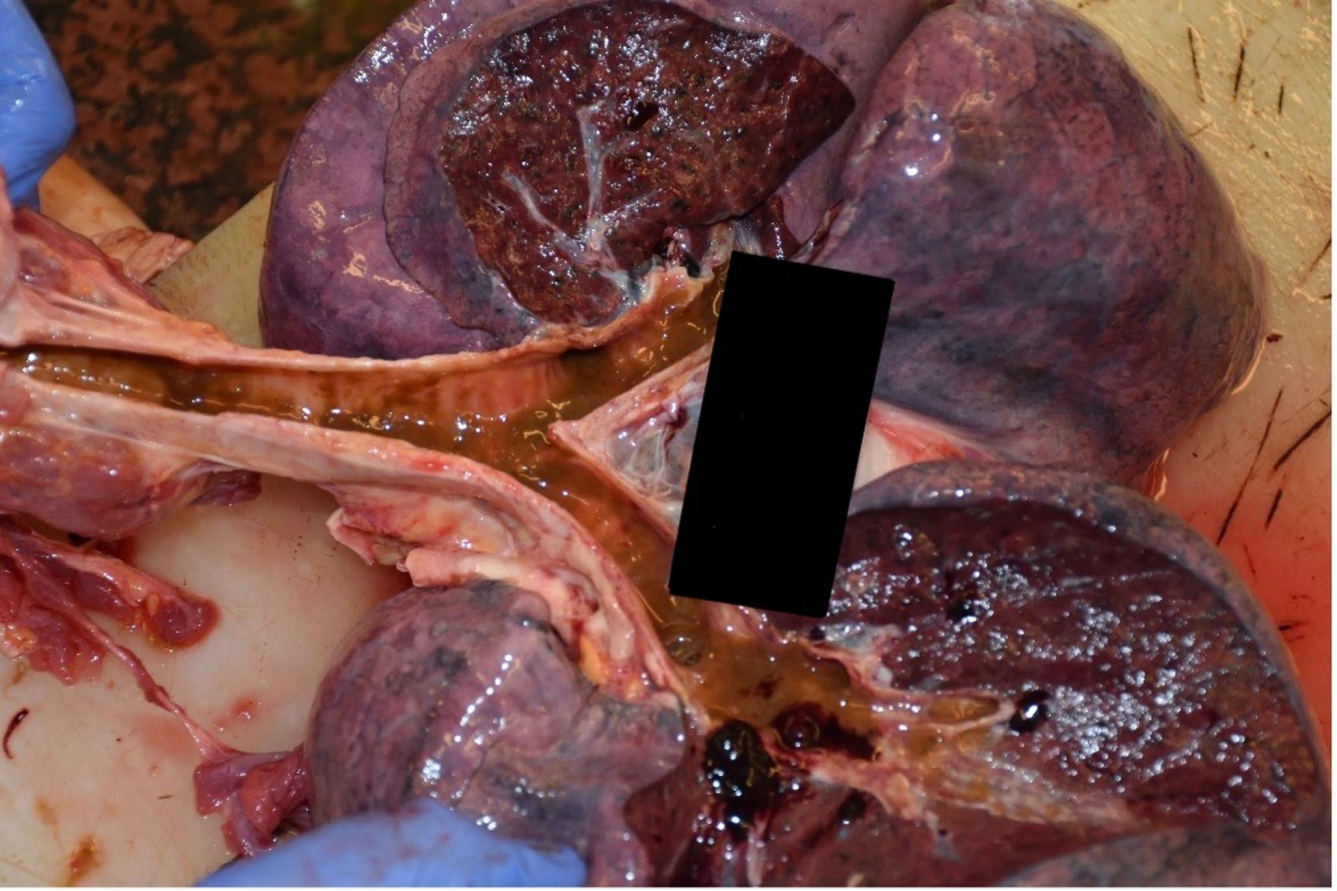
Evidence of Airway Obstruction Due to Soil Aspiration. The image illustrates the presence of soil particles within the airway, strongly suggesting inhalation prior to death. The obstruction of the respiratory tract by these foreign materials likely contributed to asphyxiation, leading to fatal respiratory failure. The distribution and depth of the aspirated soil indicate that the victim was still breathing when the material entered the airway. These findings support the conclusion that suffocation occurred due to airway compromise caused by soil aspiration

**Fig. 4:**
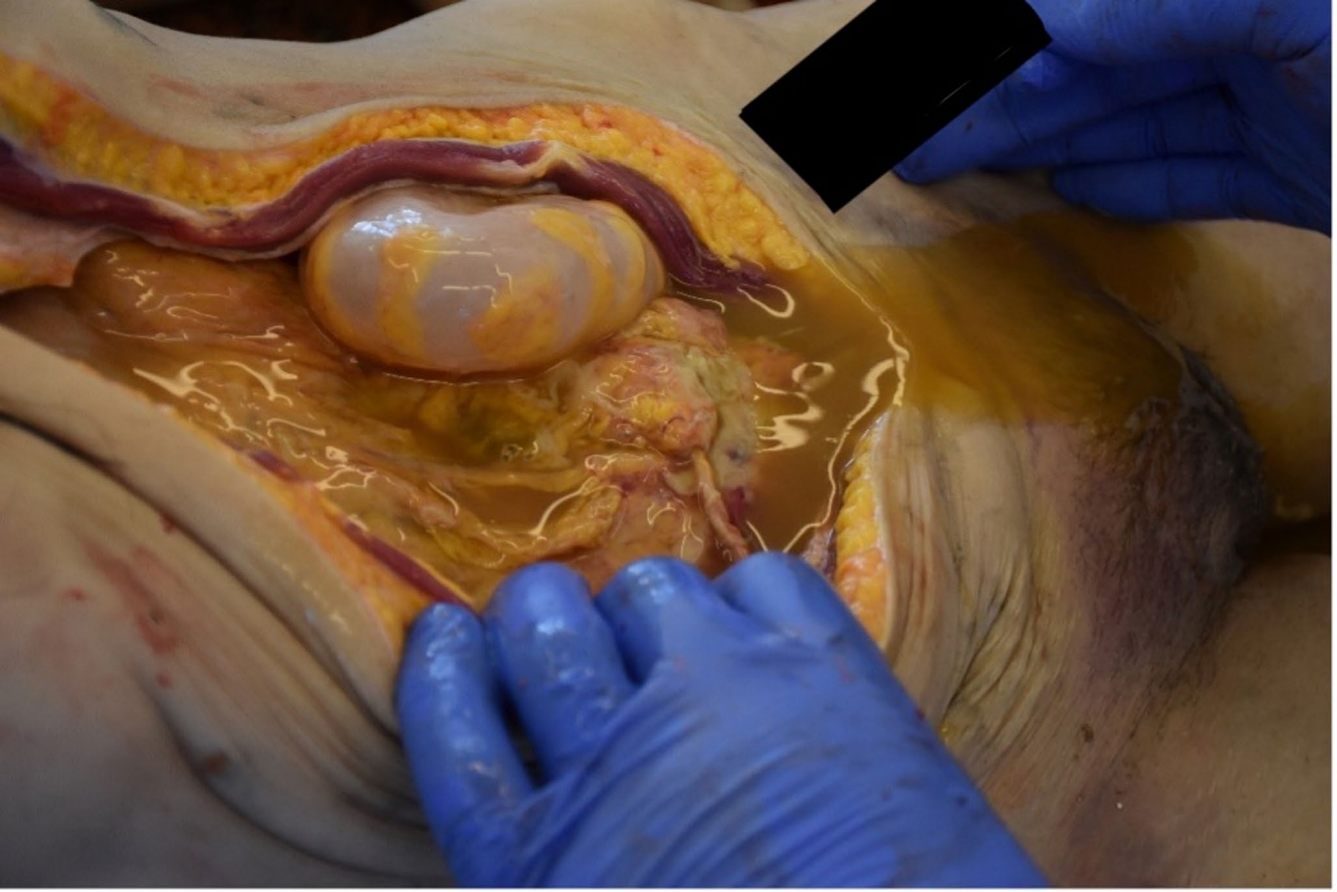
The Perforated Duodenum. Exposed duodenum with an approximately 10 mm ulcerative perforation. The lesion exhibits well-defined margins with evident tissue necrosis, indicative of a chronic peptic ulcer. The surrounding mucosa appears inflamed, reinforcing the presence of an ongoing pathological process. Given the location and characteristics of the perforation, it likely contributed to the previously observed peritonitis and intra-abdominal fluid accumulation

**Fig. 5:**
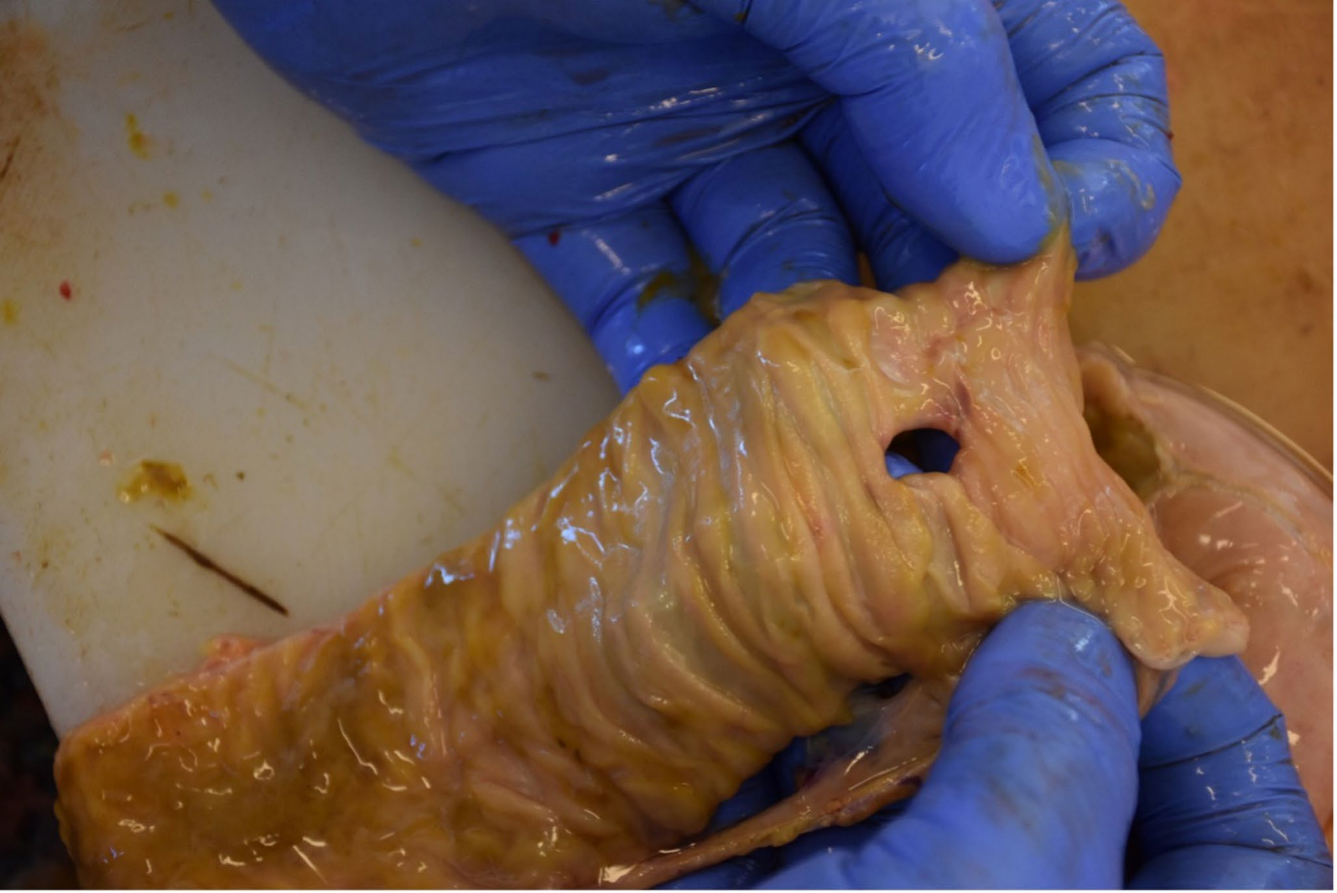
Peritonitis and Intra-abdominal Fluid Accumulation. Upon examination of the abdominal cavity, a significant accumulation of inflammatory fluid was observed within the peritoneal space. The peritoneal membranes displayed clear signs of inflammation, including diffuse erythema, thickening, and fibrin deposits. Notably, a 10 mm diameter perforation was identified, which is likely the source of the peritonitis and intra-abdominal fluid accumulation. Despite the presence of peritoneal inflammation, no visible food particles or undigested material were observed within the peritoneal cavity, suggesting a contained or partially sealed perforation. These findings indicate a severe intra-abdominal inflammatory response, with the potential for systemic complications if left untreated

**Fig. 6:**
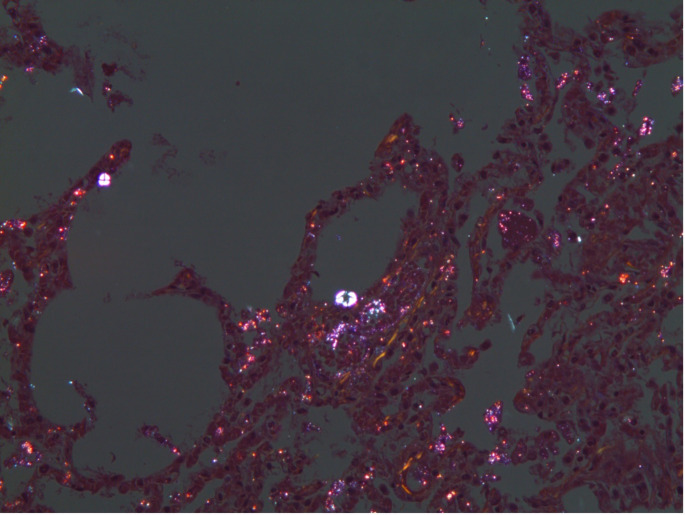
Lung Tissue Section Examined Under Polarized Light Microscopy. The image presents a histological section of lung tissue analysed using polarized light microscopy. Light-refracting soil-derived particles are distinctly visible, adhering to the alveolar walls and dispersed within the pulmonary parenchyma. The presence of these foreign particles deep within the lung tissue strongly suggests aspiration of soil material ante-mortem. This finding supports the hypothesis of airway obstruction due to soil inhalation, which likely contributed to asphyxiation. The distribution of particles within the alveolar spaces further indicates that the victim was still breathing at the time of exposure to the foreign material, reinforcing the conclusion that suffocation played a central role in the cause of death

A 32-year-old woman was admitted to the Department of Forensic Medicine for a medico-legal autopsy. According to the police report, a middle-aged man was repeatedly violent towards a partner. In the testimony, the man stated that the partner had been feeling unwell for a few days. One afternoon, feeling very weak the victim went to bed. Upon returning of chopping wood, the man noticed that the other person was not moving. In a state of panic at the possibility that the partner was dead, the perpetrator buried the body in the shed (Fig. 1). Externally, the body of the young woman measured 158 cm in height and appeared well-developed and well-nourished. The skin exhibited widespread contamination with soil, particularly on the head and upper limbs. Post-mortem hypostasis was present, and rigor mortis persisted in the lower limbs. The skull was regular in shape, and the hair was dark brown, measuring approximately 7–12 cm in length. Soil contamination was observed around the mouth, ears, and nose, but no fresh petechial haemorrhages were identified on the skin of the deceased. Additionally, non-fresh haemorrhages were noted on the scalp, with scattered haemorrhages in the mid-facial region. The nose showed signs of deformity. Multiple bruises and abrasions were distributed across the body, indicating injuries of varying ages. Internally, watery brain swelling was observed, along with an incarceration groove on the cerebellar tonsils. The lungs were swollen (Fig. 2), and the airways contained sandy material (Fig. 3). There was also evidence of bone scarring on the 9th and 10th ribs on the left side. Furthermore, a chronic perforated ulcer (10 mm of diameter) was present in the small intestine (Fig. 4), leading to peritonitis and intra-abdominal fluid accumulation (Fig. 5). No head trauma or other bone fractures were observed. A toxicological investigation detected no alcohol or drugs in the blood sample. Histopathological examination revealed acute emphysema. Soil particles were identified by polarization microscopy investigating the airways and the lungs (Fig. 6). It was concluded that the true cause of death was suffocation due to being buried alive, revealing a tragic misjudgement and its fatal consequences.

## Discussion

This is the first case in the literature reporting that a severely ill patient can be buried alive without protest and without the need for any coercive measures, such as restraint or pharmaceutical/alcoholic influence. Death likely occurred quickly after alive burial due to the pathological condition, as only trace amounts of soil residue were observed in the airways. However, it was detectable using polarized light microscopy. The victim was positioned close to the ground surface at the burial site, making asphyxiation due to compression less likely.

Asphyxiation due to sand aspiration is an uncommon phenomenon, particularly in homicide cases, as it is primarily associated with accidental occurrences [3, 6, 7].

In forensic literature, such incidents typically involve workplace hazards or environmental factors rather than intentional acts of violence. However, when suspected in a criminal case, a detailed forensic investigation is essential to distinguish between accidental and deliberate causes of death.

One documented case involves a workplace accident where an individual was buried under a load of sand unintentionally dumped by a wheeled loader shovel. Initially, witnesses suggested that the death was of natural origin, but the forensic pathologist identified inconsistencies and reported the case to law enforcement. The presence of sand in the deceased’s eyes, mouth, and airways, as well as radiological evidence, indicated asphyxiation due to sand inhalation [7]. This case underscores the necessity of meticulous forensic analysis in seemingly non-suspicious deaths, as medical evidence was instrumental in correcting the initial misdiagnosis and prompting a police investigation into the true circumstances.

Another notable case highlights the dangers associated with beach sand collapses, where victims suffered fatal asphyxia due to chest and neck compression rather than direct airway obstruction by sand aspiration. Autopsy findings, including petechial haemorrhages, cervical congestion, and minor haemorrhages in the neck muscles, provided crucial forensic indicators of asphyxiation caused by external pressure [3]. This case illustrates the importance of raising public awareness about the risks of unstable sand structures and emphasizes the need for preventive measures to mitigate similar fatal accidents in the future.

In the present case, forensic and medical analyses played a critical role in determining both the cause of death and the level of responsibility of the suspect. The perpetrator had previously subjected the victim to repeated physical abuse, as documented in medical reports. The post-mortem examination revealed evidence of prior injuries in various stages of healing, further corroborating a history of assault. Upon opening the abdominal cavity, purulent inflammatory exudate was immediately noted, indicative of peritonitis. Examination of the gastrointestinal tract revealed a 10 mm perforation in the duodenum, consistent with a perforated ulcer. The resulting peritonitis constituted a severe, potentially fatal condition in the absence of timely medical intervention. It is plausible that the perpetrator misinterpreted the victim’s condition as death.

Historically, such misjudgements led to documented cases of premature burial, particularly before the 19th century, when diagnostic limitations often resulted in individuals being mistakenly pronounced dead. This widespread fear (taphophobia) even gave rise to the development of safety coffins equipped with signalling mechanisms, such as bells or breathing tubes, allowing the mistakenly buried to alert those above ground [5, 8]. Frédéric Chopin, concerned that the determination of death might be inaccurate and vivisepulture could result, requested the removal of the prior to burial in order to preclude such a possibility [8]. This decision was not solely driven by the widespread contemporary fear of premature burial, which stemmed from the diagnostic limitations of the time, but also bore profound symbolic significance. By arranging for the return of the heart to his homeland, Poland, a lasting connection was established with the country to which Chopin remained deeply devoted throughout life, despite spending much of it in exile [8, 9].

In the present case, believing the victim to be deceased, the perpetrator acted in a state of panic and disposed of the body in an inappropriate manner. The remains were placed in a shallow grave within the shed and covered with soil. However, the subsequent autopsy revealed the presence of soil particles within the airways, providing definitive evidence that the victim was still alive at the time of burial, albeit likely unconscious.

The most pivotal forensic discovery was that the victim was still alive at the time of burial. The autopsy revealed the presence of soil particles deep within the trachea and lungs, a strong indication that the victim had been breathing and struggling for air while buried. This ruled out the possibility that the deceased had succumbed to other causes prior to burial and directly linked the suspect’s actions to the death. Additionally, forensic toxicology analyses excluded the presence of drugs or alcohol that might have contributed to the collapse, further reinforcing the conclusion that death resulted from mechanical asphyxiation.

In cases of live burial, whether resulting from an accident or committed with homicidal intent, a coffin is not used, making the cause of death dependent on at least two factors. Firstly, it is evident that soil or sand entering the mouth and subsequently the airways lead to asphyxiation. However, the thickness and weight of the overlying soil or sand must also be considered. So, secondly the avalanche-like landslides or sand collapses often cause death through this latter mechanism, as several tonnes of weight may compress the chest, rendering respiratory movements impossible [2]. Moreover, this can occur even when the entrances to the airways (nose and mouth) remain unobstructed.

Burying someone alive with homicidal intent is only possible if the victim is incapable of resisting, for instance, if they are bound [5] or under the influence of medication, sedatives, general anaesthetics, or alcohol. The peculiarity of the present case lies in the fact that the victim was unable to resist live burial due to a medical condition and no drug or alcohol were identified in the deceased. The peritonitis associated with duodenal perforation had caused such a severe pathological state that the victim was barely conscious - perhaps even entirely unconscious. This state of unconsciousness misled the perpetrator into believing the victim had already died, leading to the inadequate decision to bury alive in the shed, which ultimately resulted in death. It is highly probable, though not certain, that the victim would have succumbed to peritonitis regardless. However, in the absence of appropriate medical intervention, this outcome would have been also a conceivable scenario.

This case exemplifies the fundamental role of forensic pathology in distinguishing between intentional and unintentional deaths in criminal investigations. The presence of soil in the respiratory system was a key piece of evidence demonstrating that the victim had not been deceased before burial. Rather, it confirmed that a prolonged and distressing form of asphyxiation had occurred due to being buried alive. The forensic findings in this case not only clarified the sequence of events but also highlighted the tragic misjudgement of the perpetrator, ultimately shaping the legal and investigative outcomes of the case.

## Conclusions

This case underscores the essential role of forensic medicine in uncovering the truth behind suspicious deaths. The autopsy findings transformed what initially seemed like an abuse-related death into a criminal investigation, demonstrating how forensic pathology helps in distinguishing between accidental and intentional deaths. This highlights the importance of thorough forensic examination in cases where the cause of death is uncertain, ensuring that justice is served based on scientific evidence. This case highlights that certain severe illnesses can lead to such extreme physical and mental exhaustion, as well as indifference, that the affected individual can be buried alive without resistance or protest, without the need for any coercive measures, restraints, or the use of mind-altering substances. This suggests that critically ill patients may reach a level of vulnerability where they do not actively respond even to a life-threatening situation.

### Key points

1. Burial in soil may lead to fatal mechanical asphyxia through airway obstruction or chest compression.
2. In rare cases, severely ill individuals may be buried alive without resistance or drug influence.
3. Soil particles detected deep in the airways confirm respiration during burial.
4. Forensic pathology was essential in distinguishing live burial from post-mortem concealment.
